# The profile of illegal advertising of tobacco and nicotine products on social networks in Brazil

**DOI:** 10.18332/tpc/207095

**Published:** 2025-08-29

**Authors:** Breno M. Abreu, Raiane D.N. Assimos, Larissa M. Ricardino, Juliana D.S. Frazão, Stefania S. Piras, Patricia A. Castello Branco, Patricia G.D. Albertassi, Jeanne A.V. Cavaggioni, Ana Márcia M.S. Fernandes, Andre Luiz O. da Silva

**Affiliations:** 1 Agência Nacional de Vigilância Sanitária, Brasília, Brazil; 2 Instituto Federal de Educação, Ciência e Tecnologia do Rio de Janeiro, Rio de Janeiro, Brazil; 3 Universidade Federal Fluminense, Niterói, Brazil

**Keywords:** social networks, tobacco advertising, tobacco industry strategies, electronic cigarette advertising

**Dear Editor,**

The marketing of tobacco and nicotine products is one of the key drivers for the use of these products^1^. As a result, many countries have adopted restrictions on the advertising of these products, and the WHO Framework Convention on Tobacco Control (FCTC) and its guidelines also make recommendations for the marketing of these products to be banned. In 2021, 91 countries had some kind of restriction on the advertising of tobacco items^2^. The internet has been a particularly challenging place for regulatory authorities, and literature indicates that a significant proportion of young people are exposed to online content relating to tobacco products and nicotine^3^.

In Brazil, Instagram is the 2nd most used social network (only behind WhatsApp) with around 135 million users^4,5^. Facebook is the 3rd most accessed social network by Brazilians, with around 112 million users, and has a profile of users in older age groups^4,5^.

In Brazil, the advertising and sale of tobacco and nicotine products on the internet are prohibited. Therefore, the aim of this study was to evaluate the nature of tobacco and nicotine product advertisements on Instagram and Facebook.

Within this cross-sectional study, search data were used to verify compliance, as part of the inspection activities of ANVISA (Agência Nacional de Vigilância Sanitária) from 1 July to 30 September 2024, the URLs available on Facebook and on Instagram, regardless of publication date (Supplementary file). During the search, the URLs found were defined as legal or illegal according to the law. Illegal URLs were removed. The assessment of a URL’s legality was based on the identification of a specific brand or brand family, either through textual references or visual representations. URLs containing such identifiable branding elements were classified as illegal. Conversely, URLs depicting individuals using products were deemed legal, provided that no specific brand could be recognized. For the purposes of this analysis, the term ‘URL’ refers to individual posts published within a given account. The URLs were initially identified and coded by one author and subsequently reviewed by a second. In instances of disagreement, a third author was consulted to adjudicate and reach a consensus.

Illegal URLs were categorized (coded) according to the product advertised and the nature of the advertisement as follows: sale of products (where prices and purchase contacts are provided, even if accompanied by any other elements); display of products (posts that merely showcase the products, without including any promotional text, pricing information, contact details for purchase or any other elements); use or instruction in the use of the product (cases where the URL had images of people using or teaching how to use the product); brand promotion (where only the brand logo is displayed, without any accompanying product image or other element); social performance/sexuality (associated with elements of financial, social, sexual, or romantic success); health benefits or harm reduction, association with films (this includes non-human characters, movie posters, and actors portrayed as specific characters, assuming no additional elements such as pricing information are present); cartoons (including cartoon elements on the packaging is acceptable, with no other elements such as pricing information, health claims, or references to social performance); celebrities (including actors, but not characterized as a specific character); and sports (including sport elements on the packaging is acceptable, with no other elements such as pricing information, health claims, or references to social performance).

The monitoring carried out found 5941 URLs on both networks during the period studied. Of these URLs, 5930 were on Instagram and 11 on Facebook. All pages were removed after ANVISA’s determination (Figure 1). With regard to the products advertised, 2993 were EDS (Electronic Devices to Smoke, definition that includes e-cigs and HTPs), and 2948 were conventional tobacco products. Among the conventional tobacco products, 2020 were hookah tobacco, 261 were shredded tobacco, 242 were straw cigarettes, 206 were more than one product type in the same advertisement, 130 were cigars, 5 were kreteks, 24 were cigarettes, 5 were snuff, 3 were chewing tobacco, 1 was little cigars.

**Figure 1:**
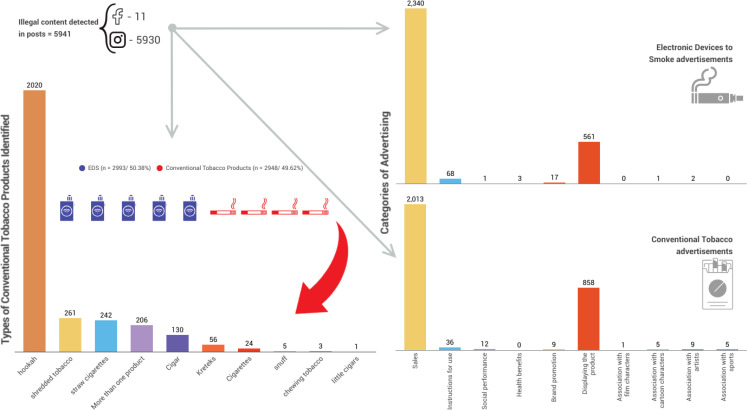
Profile of tobacco and nicotine products advertising on social media in Brazil

Among conventional tobacco products, the marketing advertising elements found were: 2013 items aimed at direct sales, 858 items aimed at product display, 36 items containing instructions for use or consumption of the product, 12 items aimed at social performance/sexuality, nine items aimed at brand promotion, nine aimed at advertising associated with artists, five items associated with cartoon characters, five items associated with sports, and 1 item associated with film characters (Figure 1).

Among EDS, the advertising elements found were: 2340 aimed at selling products, 561 aimed at displaying the product, 68 aimed at instructions for consumption of the product, 17 aimed at promoting the brand, 3 containing information about harm reduction/health benefits, 2 in association with artists, 1 containing better social performance/sexuality, and 1 associated with a cartoon character (Figure 1).

Our results indicated that nicotine and tobacco product advertisements in Brazil are significantly more widespread on Instagram than on Facebook. The higher number of advertisements on Instagram than on Facebook can be explained for a number of reasons, ranging from the greater number of Instagram users compared with Facebook in Brazil, to the nature of the platform, which focuses on images and short videos, and the profile of users, who are younger.

There were also a large number of illegal advertisements for EDS. This high number of advertisements on the internet could be explained by the ban on the sale of these products and the fact that their target audience is mostly young people^3,4,6^. This can be confirmed by the large number of URLs dedicated to the sale of these products and their exposure. Also noteworthy are the URLs dedicated to giving instructions on how to use these products, which seem to be aimed at inexperienced users and possibly those who have never used the product before. Comparatively few URLs were found pointing out health benefits and association with cartoon characters. Regarding conventional tobacco products, hookah was the item most found, followed by shredded tobacco and straw cigarettes. In the case of hookah, the data are in line with epidemiological data showing that these products have appeal among young Brazilian adults^7^. Straw cigarettes are a tobacco product in which corn straw is used to roll the tobacco. They are mostly consumed in states with a predominantly rural economy in Brazil^8^. As for the nature of the advertising for conventional tobacco products, websites encouraging direct sales accounted for the largest number of URLs (posts) found, followed by URLs advertising brands, which could be explained by the ban on all forms of advertising for tobacco products, and by the fact that the brands found were not registered with ANVISA.

Although comparatively less prevalent when compared to the other advertising elements, the use of cartoon characters and characters with better social performance (status, better capacity for interaction, sociability, sexuality, and attractiveness) and the decrease in perceived risks are a cause for concern, as these are elements that particularly engage with children and adolescents^9,10^.

The illicit market and illegal advertising, due to their nature and their rapid changes to evade coercive mechanisms, pose a challenge for monitoring these activities, making them difficult to search for, which can limit searches and proper monitoring. Only two social networks were searched, so the results found here are limited to the time frame used and to Facebook and Instagram only. Another limitation involves the algorithmic architecture of social media platforms, which are not designed to facilitate comprehensive or systematic content searches, and the inadequacy of their native search functions presents a significant challenge for researchers. As a result, it is likely that some relevant posts were not captured during data collection, potentially limiting the breadth and representativeness of the findings,

This is the first study to investigate the advertising of tobacco and nicotine products on social media platforms in Brazil. The prevalence of content targeting younger audiences aligns with international findings^6^. Although both platforms officially prohibit paid tobacco advertising, these policies have proven inadequate in preventing such content. Thus, it is crucial to discuss regulatory strategies and enforcement mechanisms that can more effectively address and restrict this form of advertising, especially given that the internal policies of social media platforms have been shown to be insufficient. Notably, ANVISA and Meta group made an agreement to facilitate the enforcement of advertising restrictions. The large number of requests from ANVISA to remove illegal URLs with unauthorized advertisements for various products like medicines, food, and tobacco, prompted the Meta group to establish a hotline dedicated to the removal of these URLs. This cooperation agreement with the social networks was an important element in getting the illegal URLs removed, which is a public health gain.

## Supplementary Material



## Data Availability

The data supporting this research cannot be made available for privacy or other reasons.
