# Supplementary material for: The profile of illegal advertising of tobacco and nicotine products on social networks in Brazil
Source: Tob Prev Cessat. 2025 Aug 29;11:10.18332/tpc/207095. doi: 10.18332/tpc/207095 (PMC12395513; doi:10.18332/tpc/207095)

Supplemental file

THE PROFILE OF ILLEGAL ADVERTISING OF TOBACCO AND NICOTINE PRODUCTS ON SOCIAL NETWORKS IN BRAZIL

Tobacco Prevention and Cessation

List of search terms used:

- Bidi
- Blunt
- Box mod
- Chewing tobacco
- Cigar
- Cigarette
- Cigarillo
- Disposable electronic cigarettes
- E-cigarette / E-cig
- E-cigs
- E-hookah
- Electronic Device to Smoke (and the acronym in portuguese DEF)
- E-liquids
- ENDS
- ENNDS
- E-pod
- E-shisha
- Hand-rolled cigarette
- Heated tobacco
- Heated Tobacco Product
- Heat-not-burn
- HTP
- Mod
- Pipe tobacco
- Pod
- Pod system
- Roll-your-own tobacco
- Shisha tobacco
- Shredded tobacco
- Snuff
- Stick
- Stick vape
- Straw cigarette
- Tank
- Vape pen
- Vapes
- Vaporizers

Some examples of URLs found (To ensure confidentiality and maintain the integrity of the investigations, some details have been intentionally redacted):

Product – Disposable vapes product

Advertisement type: Social performance sexuality - associating tobacco/nicotine products with nightlife, glamour, and social appeal.

Status: Illegal

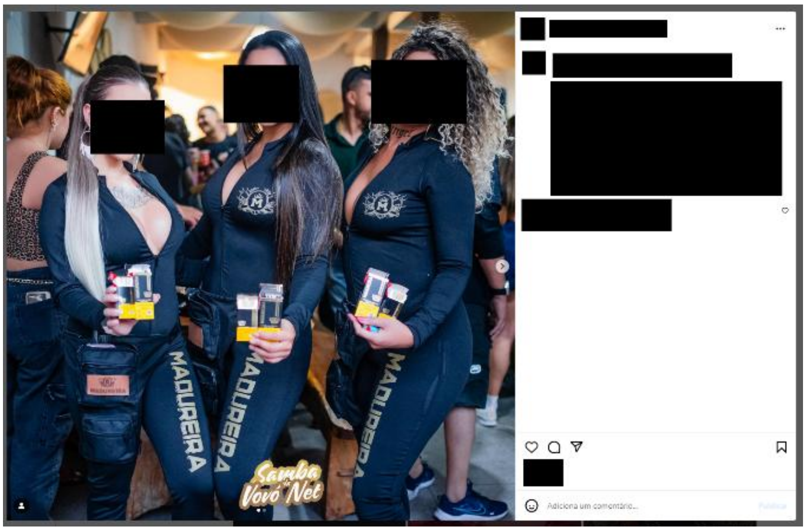

Product: E-cigs (e-liquids)

Advertisement type: Sales (price included in the post)

Status: Illegal

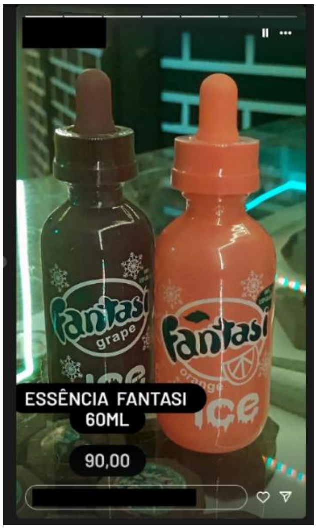

Product: Shisha tobacco

Advertisement type: Association with sports (football)

Status: Illegal

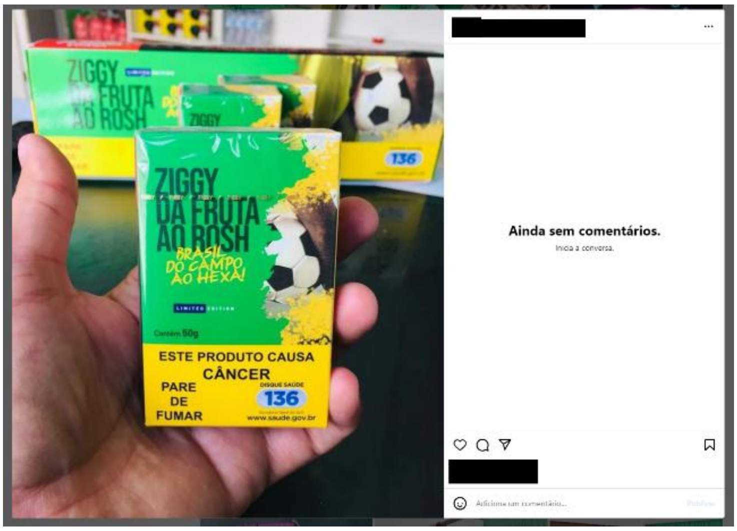

Product: Shisha Tobacco

Advertisement type: Association with movies (characters and films from the horror genre)

Status: Illegal

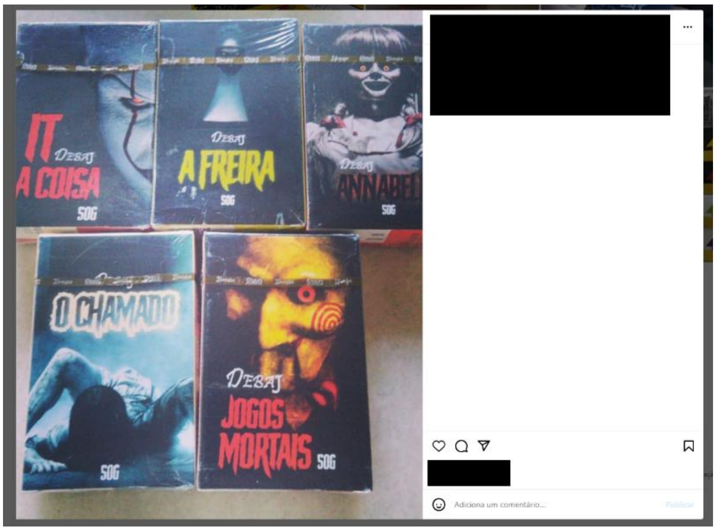

Product: Electronic cigarettes

Advertisement type: Health benefits (Message suggesting that e-cigs save lives)

Status: Illegal

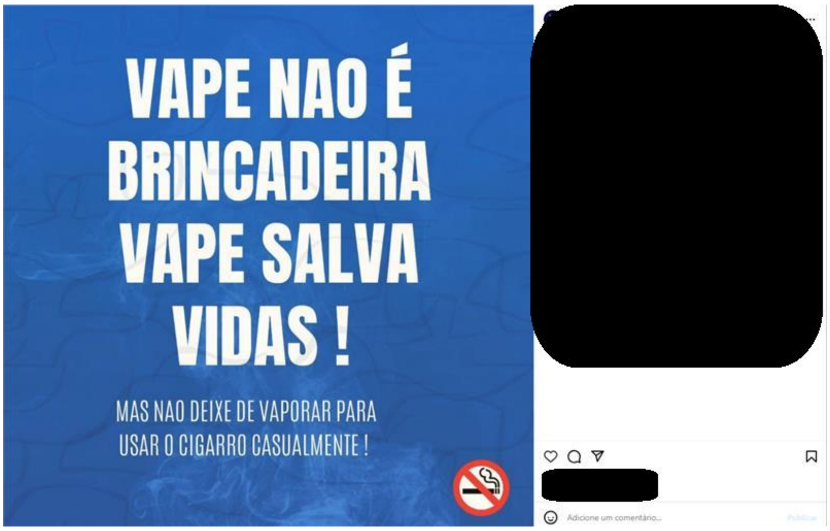

Product: Cigars

Advertisement type: Product display (a specific brand of cigar being displayed, with no additional elements present)

Status: Illegal

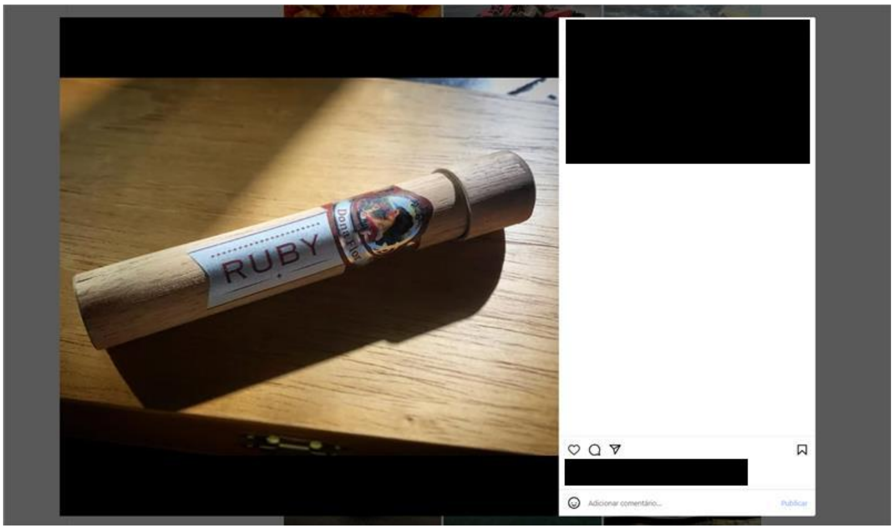

Product: Shisha tobacco

Advertisement type: Association with cartoons (various cartoon characters featured on the packaging)

Status: Illegal

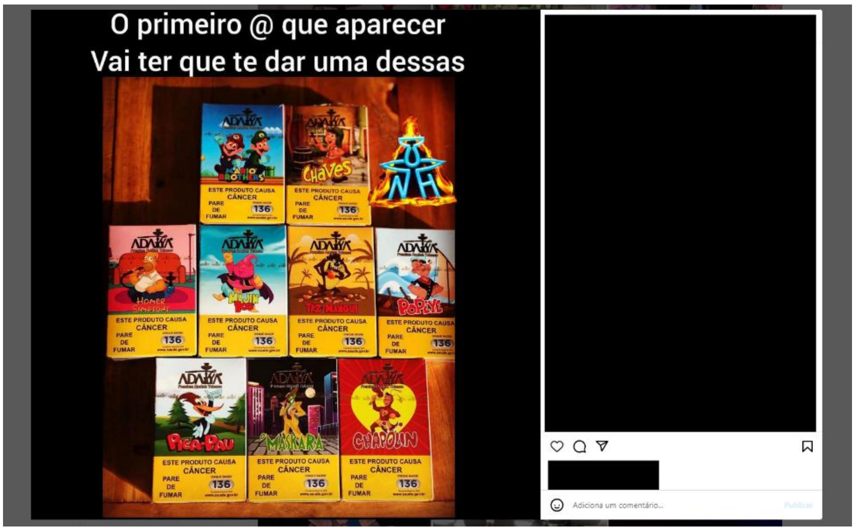

Product: Shisha tobacco

Association with cartoons type: This does not qualify as advertising for tobacco or nicotine products (no specific brand or tobacco/nicotine company was mentioned)

Status: Legal

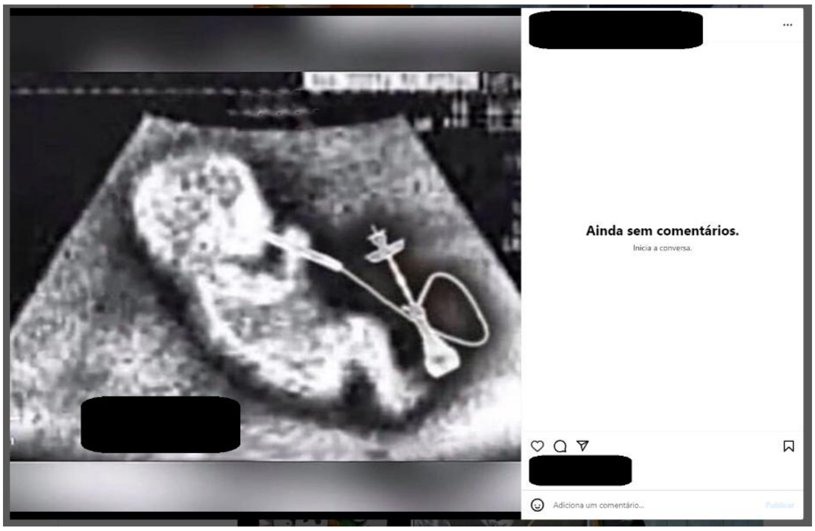

Supplement: Supplementary file 1 [file TPC-11-40-s1.pdf]
